# An analysis of transition-resulted goal scoring patterns in football leagues: a comparison of the first 5 rounds and the last 5 rounds prior midway of the season

**DOI:** 10.1186/s13102-024-00854-0

**Published:** 2024-03-02

**Authors:** Pedro Eusebio, Pablo Prieto-González, Rui Marcelino

**Affiliations:** 1 Sport Sciences and Diagnostics Research Group, GSD-HPE Department, Prince Sultan University, Riyadh, 11586 Saudi Arabia; 2 Research Centre in Sports Sciences, Health Sciences and Human Development, CIDESD, CreativeLab Research Community, Vila Real, Portugal; 3 Sports Sciences Department, University of Maia, Maia, Portugal; 4 Portugal Football School, Portuguese Football Federation, Oeiras, Portugal

**Keywords:** Playing patterns, Offensive, Defensive processes, Offensive transition, Game moments, Tactical strategies, Soccer

## Abstract

This study investigated the concept of ‘game style’ in football, focusing on a team’s distinctive approach characterized by tactics, formations, and strategies. The objective is to compare goal-scoring patterns related to “non-offensive transition,” “set pieces,” “offensive transition,” and “positive outcomes” across different league groups. Methodology: The sample comprises 702 football games, categorized into three league groups: Top Leagues, Marginal Leagues, and Emerging Leagues. A total of 2140 goals were analyzed, classifying them into four types: “No Transitions”, “Set Pieces”, “Offensive Transitions (direct), and “Positive Outcomes” of offensive transitions. The study aims to identify relevant differences between two distinct competitive periods, namely “Rounds 1 to 5” and “Rounds Last 5.” Results: The analysis reveals no significant differences in scoring methods between “Rounds 1 to 5” and “Rounds Last 5” across all variables in the analyzed league groups, except for goals conceded by “No Transition” in the Emerging Leagues. Additionally, results indicate that goals resulting from direct offensive transitions and positive outcomes substantially impact the Top Leagues, constituting 54% of the total scoring. Conclusion: The study emphasizes the importance of establishing and maintaining consolidated game patterns, as demonstrated by the consistent scoring patterns across different league groups. While most leagues show no variations in scoring methods between early and late rounds, attention to goals conceded by “No Transition” in Emerging Leagues is warranted. The prominence of direct offensive transitions and positive outcomes in Top Leagues highlights the relevance of these strategies in the scoring process, emphasizing the need for teams to focus on developing and implementing effective game patterns.

**Supplementary Information:**

The online version contains supplementary material available at 10.1186/s13102-024-00854-0.

## Background

The concept of *game style* is a commonly used term in sports to describe a team’s distinctive pattern of play during games. In top-level football, the identification of a team’s playing style or patterns of play has significant practical implications. These include the selection of players who are capable of fitting into and adapting to a particular style [1] and the hiring of coaches who align with the club’s culture and vision for how the team will play in future games. To better understand the repeatability and predictability of play patterns of different teams from game to game [2], numerous tactical performance indicators have been proposed [3–5] as well as tactical metrics [6], and tactical patterns [4].

According to Hewitt and collaborators (2016) the definition of game style is the characteristic of the game pattern most evidenced by a team during the games. It will be regularly repeated in specific situational contexts such that measurement of variables reflecting game style will be relatively stable.” (p. 367). The same authors conclude that “variables of importance are player and ball movements, interaction of players, and will generally involve elements of speed, time and space (location)”. (p. 367) Thus, the game patterns are considered separately in three distinct phases of the game: Established Offense and Defense, Transitional Play and Set Pieces [2].

Different intervenient (coaches, players, and researchers) consider the offensive/defensive transitions moments, as the two most important moments in football nowadays [7]. Praising that different studies point to the offensive transition as the key moment in today’s football, which has gained increasing importance at the final result of the matches [6–9] the performance analysis of game patterns gain extraordinary importance.

The understanding of a team’s game patterns especially in the transitive phases, whether it be their own team or opposing teams, has a significant impact on player performance, from training to actual games. The aim is to find a balance that allows for exploiting and controlling the opponent’s weaknesses in accordance with their style of play [10]. It is evident that the style of play adopted by a team in these transition moments will affect the number and quality of scoring opportunities during a match. It is crucial to include in their tactical-strategic plan specific attack combinations that align with their style of play, as this increases the probability of success [11]. Different studies related to game patterns have been carried out, in order to know the effective offensive/defensive patterns in successful teams. Older studies referring to the World Cups in 1986, 1990, 1994, 1998, 1994 and 2010 [10] indicate that the most effective way to penetrate defenses and succeed is to present a high number of touches, many passes, dribbling and ball long possessions, which refers to the characteristics of the continued attack. However, more recent studies UEFA 2016 and 2015-16 English Premier League [12, 13] demonstrate that successful game patterns appear to be shorter offensive patterns, where possession is achieved in more advanced field areas, characteristic of the offensive transition. This fact becomes more important as the patterns tend to occur consistently not only among a game moment but also across games [14].

A clear strategic-tactical definition will allow greater objectivity in action, in order to disrupt the equilibrium of the opposing team [14] and consequently a greater understanding of the game by all game agents [2]. The performance analyzes make it possible to understand past performances as well as predict future patterns of behavior [15] and performances [16]. The best ranked teams are interested in adopting strategies that allow them to take advantage of transition moments [17] since they recognize in these moments advantages for obtaining a goal directly as well as a way of conditioning the opponent’s strategy.

Understanding how goals are scored and whether there throughout of a sporting season can provide coaches with valuable insights for analyzing and determining players and teams’ behaviors. Comparing the goal-scoring patterns between different stages of a season can offer to all intervenient information about league characteristics and the culture of their club. Furthermore, this data can allow coaches to tailor training units as well as game strategies and tactical plans to maximize their team’s performance. The objective of this research is to examine the scoring patterns during the first five rounds of the season and the last five rounds prior midway of the season.

## Materials and methods

### Sample and variables

**Fig. 1:**
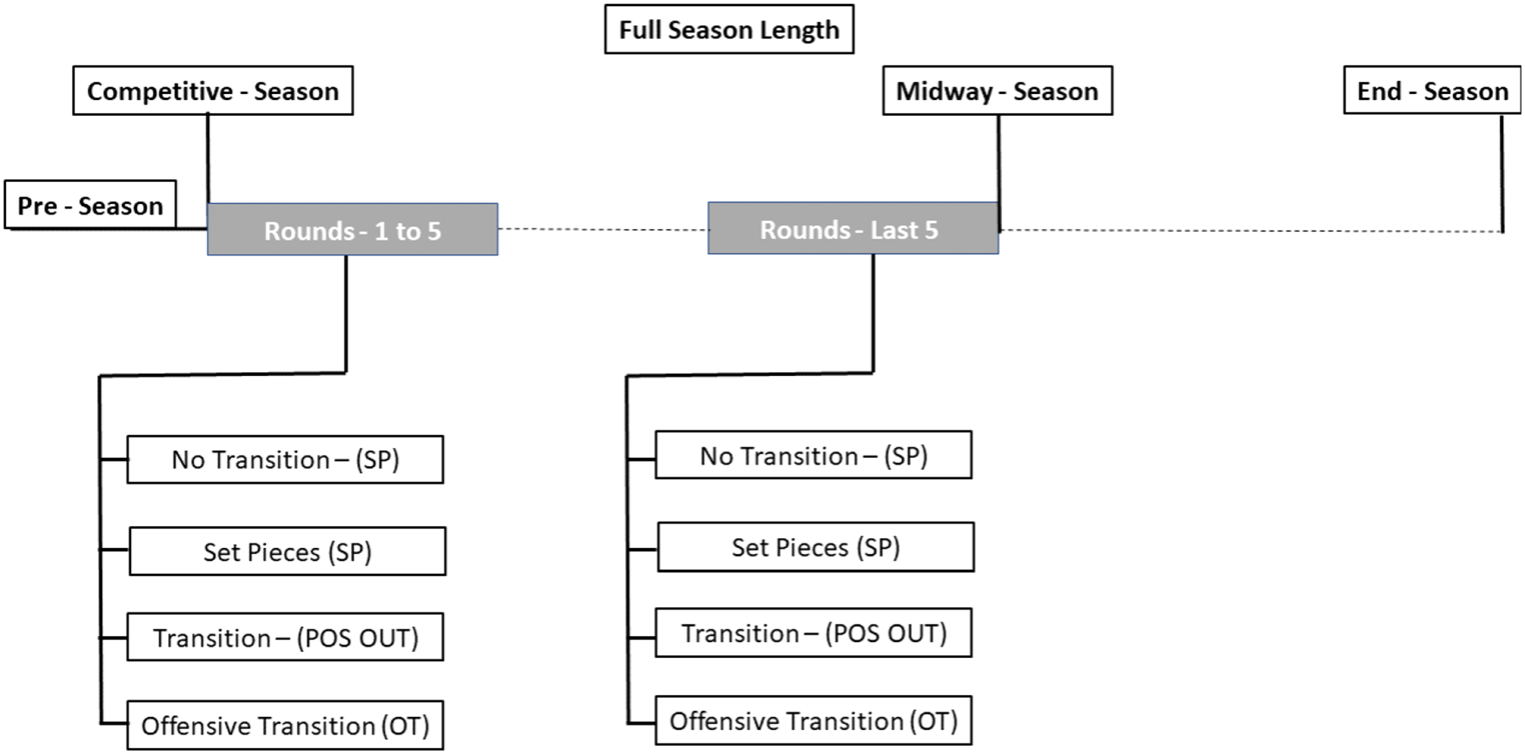
Season scope. e: The timeline shows the Full Season Length The "Rounds 1 to 5" occurs immediately after the Competitive Period starts, and "Round Last 5" take place midway of the season

The sample used for this research consisted of the 2140 goals recorded in the 702 games from the 2020/21 season. The games correspond to all matches played in the first five rounds and the last five rounds prior midway of the season in each of the nine analyzed leagues (Fig. 1). The leagues were grouped into three groups of three leagues. The Top Leagues group is made up of the Spanish, Italian and German leagues, then classified as 2nd, 3rd and 4th in the UEFA ranking (at the date the events were collected) and which are the leagues with the highest turnover and value invested in the transfer markets. The Marginal leagues, consisting of the Portuguese, Dutch and Russian leagues, which are in 6th, 7th and 8th places respectively and which present a high number of transfers from these to the others analyzed leagues and the Emerging Leagues, (EL), made up of the Qatar, Saudi Arabia, and UAE leagues that appear as markets with high financial potential that manage to attract some international players but those leagues are played mainly by local players.

All goals were categorized into four types: (i) Non-offensive Transition (NT), (ii) Direct Offensive Transition (OT), (iii) Set Pieces (SP), and (iv) Positive Outcome (POS OUT) (goals resulting from SP immediately preceding an offensive transition).

### Procedures

The instrument used for the analysis was adapted from the system proposed by Turner and Sayers (2017), along with an observation and registration instrument proposed by the authors, which is constituted by systems of categories, which precisely define the target registration criteria. The videos were collected through two video providers, InStat and WyScout. To ensure methodological rigor and comparability across teams, all games included in the analysis were restricted to the first 5 rounds and the last 5 halfway rounds of each championship. As a result, all teams were subject to an equivalent number of observations.

All plays that resulted in a goal were analyzed from the exact moment the ball was recovered until the moment the goal was scored. This includes scenarios where a team gains or regains control of the ball through a tackle, interception or rebound. In cases of quick restart of the game that led to an offensive transition, the moment of the beginning of the first action, such as throw-in, goal kick or free kick, was considered.

The goals scored by OT were described in detail. Goals classified as SP were analyzed based on the immediately previous play. If the previous play was not an offensive transition, the analysis was done succinctly. These goals maintained the classification as SP. However, if the SP was initiated by an offensive transition, the play was described in detail and classified as POS OUT. The description of these plays (POS OUT) was the same as the OT, with the end of the play being considered the moment of the positive outcome, which resulted in an immediate goal. For goals classified as NT, the same succinct description performed in SP was carried out.

### Statistical analysis

The statistical procedure used was the Estimate Independent Mean Difference, where the values of two variables (“Rounds 1 to 5” and “Rounds Last 5”) were compared according to each of the alloys analyzed (No Transition (NT), Offensive Transitions (OT), Positive Outcome (POS OUT), and Set Pieces (SP)). This procedure aimed to calculate the average the difference and confidence interval for each goal-scoring method across the three groups of leagues. All the analysis were performed by Jamovi – ESCI Software. All the images used are publicly available which means that no informed consent or ethics committee approval was required [18, 19].

## Results

A total of 2140 goals were scored in 702 games, as shown in Table 1, which displays the distribution of goals among the leagues, the goal classification and if they were scored in the first 5 rounds or in the last five rounds of the first round of the championship.

The results indicate that were scored more 38 goals were scored in “Rounds 1 to 5”. There is no noticeable difference in the number of goals scored in the first five rounds compared to the last five rounds among the group of leagues. “Rounds 1 to 5” accounted for 50.89% of the total goals scored in both variables (“Rounds 1 to 5” and “Rounds Last 5”), while “Rounds Last 5” accounted for 49.11%.

**Table 1:** Distribution of the types of goals per group of leagues on the variables “Rounds 1 to 5” and “Rounds Last 5”

			Rounds − 1 to 5				Rounds - Last 5				
	**Games Observed**	**Total Goals**	**Goals by NT**	**Goals by OT**	**Goals by SP**	**Goals by POS OUT**	**Goals by NT**	**Goals by OT**	**Goals by SP**	**Goals by POS OUT**	**% Of goals By OT + POS OUT**
Emerging Leagues	196	630	105	109	72	36	100	99	78	31	43,65%
Marginal Leagues	238	691	134	114	81	32	102	114	84	30	41,97%
Top Leagues	268	819	107	170	78	51	105	183	85	40	54,21%
Totals	702	2140	346	393	231	119	307	396	247	101	

*Note* NT: No Transition. OT: Offensive Transition. SP: Set Pieces. POS OUT: Positive Outcome OT + POS OUT: Goals obtained by Offensive transition and Positive Outcome

There are no differences in the goals obtained by Offensive Transitions (OT) between the variables “Rounds 1 to 5” and “Rounds Last 5” across any of the league groups (Table 2).

In a more detailed analysis, the results indicate that there are no differences in scoring by offensive transition across different variables in Emerging Leagues, as evidenced by a Cohen’s value of -0.10 (95% CI [-0.39, 0.18]). In Marginal Leagues, there are no differences in scoring by offensive transition across the two variables analyzed, with a Cohen’s value of 0 (95% CI [-0.26, 0.26]). Similarly, the results from Top Leagues also suggest that there are no differences in scoring by offensive transition across the analyzed variables, with a Cohen’s value of 0.09 (95% CI [-0.15, 0.33]). All the leagues that comprise this group show average values greater than 1 goal per game per OT in both variables. The results of each league regarding goals obtained by Offensive Transitions (OT) between the variables “Rounds 1 to 5” and “Rounds Last 5” can be found on table S2. The results obtained are equally valid for the analyzes carried out league by league.

**Table 2:** Estimate Ind. Mean Difference – Goals obtained by OT/NT/POS OUT/SP in different group league (EL, ML, TL) – Comparison between “Rounds 1 to 5” and “Rounds Last 5”

		Rounds 1 to 5	Rounds Last 5	d_unbiased_
Goals by OT	Emerging Leagues	1.11 (0.91; 1.32) 98	1.01 (0.82; 1.20) 98	-0.10 (-0.39; 0.18)
	Marginal Leagues	0.96 (0.79; 1.13) 119	0.96 (0.76; 1.54)119	0.00 (0,26; 0.26)
	Top Leagues	1.27 (1.09; 1.45) 134	1.37 (1.19; 1.54) 134	0.09 (-0.15; 0.33)
Goals by NT	Emerging Leagues	1.13 (0.92; 1.34) 98	0.86 (0.70; 1.02) 98	-0.26 (-0.52; -0.01)
	Marginal Leagues	1.07 (0.86; 1.29) 119	1.02 (0.81; 1.23) 119	-0.05 (-033; 0.23)
	Top Leagues	0.80 (0.63; 0.97) 134	0.78 (0.64; 0.83) 134	-0.02 (-0.26; 0.22)
Goals by POS OUT	Emerging Leagues	0.37 (0.25; 0.49) 98	0.32 (0.21; 0.42) 98	-0.09 (-0.37; 0.19)
	Marginal Leagues	0.27 (0.17; 1.52) 119	0.25 (0.17; 1.46) 119	-0.03 (-0.29; 0.22)
	Top Leagues	0.38 (0.28; 0.48) 134	0.30 (0.21; 0.39) 134	-0.14 (-0.39; 0.10)
Goals by SP	Emerging Leagues	0.73 (0.57; 0.90) 98	0.80 (0.62; 0.97) 98	0.07 (-0.21; 0.35)
	Marginal Leagues	0.68 (0.54; 0.82) 119	0.71 (0.57; 0.84) 119	0.03 (-0.22; 0.29)
	Top Leagues	1.27 (0.46; 0.70) 134	1.37 (0.50; 0.77) 134	0.07 (-0.17; 0.31)

*Note* NT: No Transition; OT: Offensive Transition; SP: Set Pieces; POS OUT: Positive Outcome; OT + POS OUT: Goals obtained by Offensive transition and Positive Outcome; M (LL; UP) n

The analysis reveals that there are differences in goals scored by “No-Transition” (NT) in Emerging Leagues across different variables, with a Cohen’s value of -0.26 (95% CI [-0.52, -0.01]). In Marginal Leagues, the results suggest that the differences in goals scored by NT are not relevant, with a Cohen’s value of -0.05 (95% CI [-0.33, 0.23]). Similarly, in Top Leagues, the analysis shows that there are no differences in goals scored by NT across the analyzed variables, with a Cohen’s value of -0.02 (95% CI [-0.26, 0.22]). The results of each league regarding goals obtained by “No-Transition” (NT) between the variables “Rounds 1 to 5” and “Rounds Last 5” can be found on table S3.

The analysis of goals resulting from a positive outcome (POS OUT) and suggests that there are no differences in goal scoring across the variables “Rounds 1 to 5” and “Rounds Last 5”. In Emerging Leagues, the analysis reveals no expected differences in goal scoring, with a Cohen’s value of -0.09 (95% CI [-0.37, 0.19]). In Marginal Leagues, the analysis shows virtually nonexistent differences, with a Cohen’s value of -0.03 (95% CI [-0.29, 0.22]). Similarly, in Top Leagues, the results suggest no differences in goal scoring by “POS OUT” across the analyzed variables, with a Cohen’s value of -0.14 (95% CI [-0.39, 0.10]). The results of each league regarding goals obtained by positive outcome (POS OUT) between the variables “Rounds 1 to 5” and “Rounds Last 5” can be found on table S4.

Regarding goals scored from set pieces (SP), there are no differences expected in Emerging Leagues in goals scored from “SP” across the variables “Rounds 1 to 5” and “Rounds Last 5”, with a Cohen’s value of 0.07 (95% CI [-0.21, 0.35]). In Marginal Leagues, there are also no differences expected in goals scored from SP, with a Cohen’s value of 0.03 (95% CI [-0.22, 0.29]). Similarly, Top Leagues do not show expected differences in goals scored from SP across the different variables, with a Cohen’s value of 0.07 (95% CI [-0.17, 0.31]). The results of each league regarding goals obtained by set pieces (SP) between the variables “Rounds 1 to 5” and “Rounds Last 5” can be found on table S5.

**Fig. 2:**
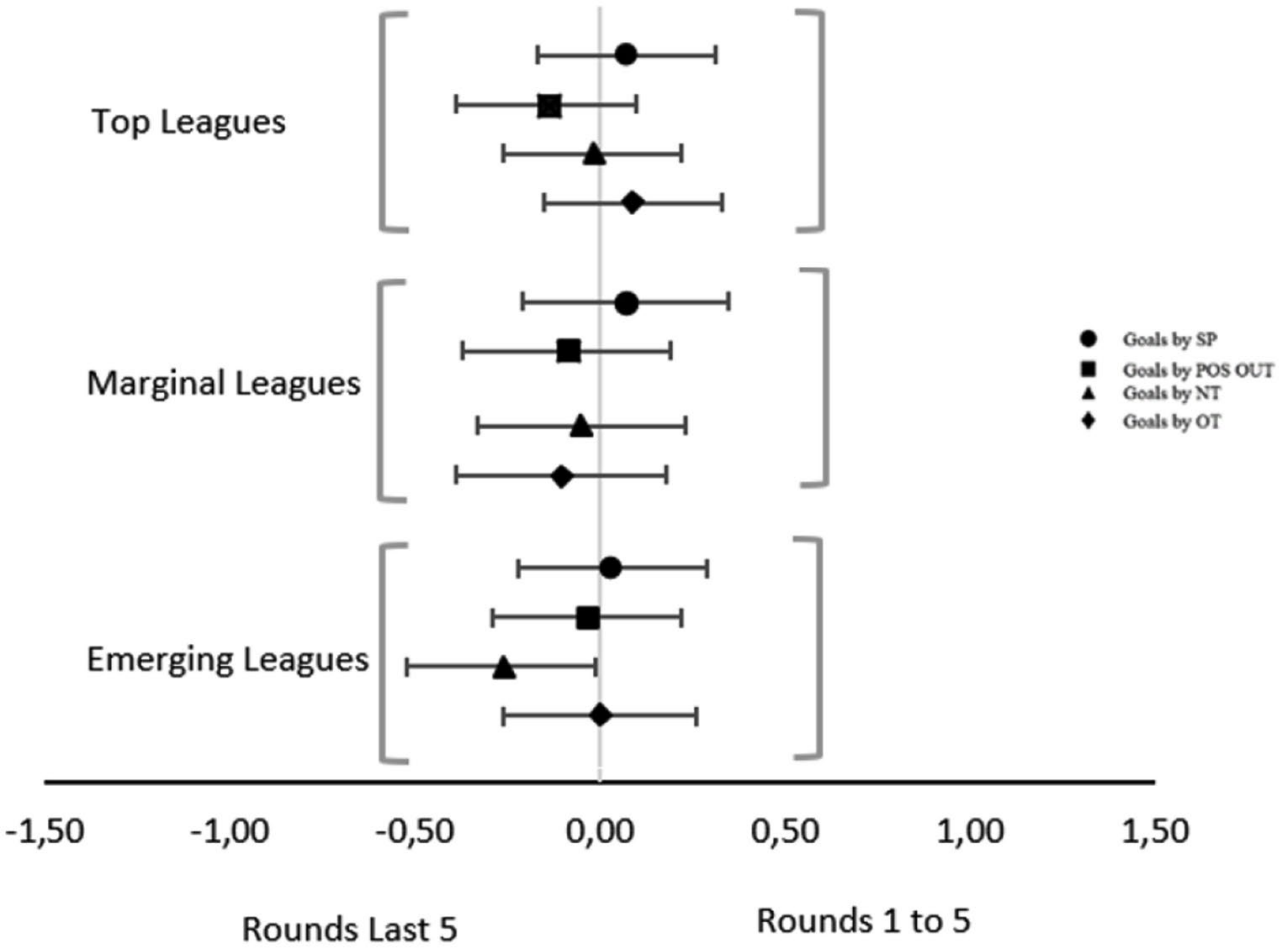
Estimate independent mean difference – Forest Plot based on ratios – leagues overview. *Note* Fig. 2 – SP – Goal by Set Pieces; POS OUT – Goals by Positive Outcome; NT – Goals by No Transition; OT – Goal by Offensive Transition.

The Fig. 2 shows the Effect Size (Cohen’s d) and the confidence intervals of the respective variables. It can be observed that these intervals have relatively similar magnitudes. Except for the “No Transition” goals scored in Emerging Leagues, all variables have their effect estimate values close to zero. Except for the previously mentioned variable, all others have their magnitude intervals crossing zero. However, it is notable that in the “No Transition” variable in Emerging Leagues, although these values have similar magnitude, they diverge from the others as zero is not included in its confidence interval.

## Discussion

The objective of this research is to compare the goal scoring patterns that result from the transitions in the first 5 rounds of the championship and the last 5 prior midway of the season in leagues that were grouped based on similarities in context sports.

The findings indicate that there are no differences in none of the method of goal scoring between the “Rounds 1 to 5” and “Rounds Last 5” variables across all the analyzed league groups. However, there was a notable exception in the Emerging Leagues where there were differences in goals scored by NT.

The goals obtained by “No Transition” (NT) are goals achieved through organized attack, which involves a slower and more deliberate positional attack [16]. This indicates a predominance of organized attack as a means of scoring goals, which will be explained by the temperatures at which the games are played. The data suggest that external factors that are characteristic of emerging leagues, such as high temperatures, explain the differences observed in the way goals are obtained between the two variables “Rounds 1 to 5” and “Rounds Last 5”. For instance, these leagues start in the last week of August, and the first variable “Rounds 1 to 5” is played until the end of September. During this period, the countries where these leagues are located experience extremely high temperatures, which explain the preference for organized attacks over more physically exhausting types of attacks as a way to score goals. The “Last 5 rounds” variable, played between the end of November and the end of January, have milder temperatures. However, teams have already done their pre-competition preparation under extreme weather conditions, which impact on their physical approach and game model during the season [20]. The organized attack also provides the involvement of a large number of players and greater time in possession searching to the best time to try to score a goal, whether due to defensive disorganization or individual action [21]. Additionally, considering the hiring history, it appears that these leagues have more signings of attacking players than defenders, which also explains the offensive supremacy of those type of attacks.

The Marginal League and Top Leagues on goals obtained by “No Transition” (NT) do not differ as the competitive period evolves which shows they are more prepared defensively and organizationally to respond to this type of attack. Although in these leagues there are a large number of athletes who play a high number of games, either for their clubs or for their national teams, the smaller number of effective training units does not remove the ability to maintain the same behavioral standards, thus demonstrating the high behavioral maturity of teams.

Regarding goals obtained through offensive transitions (OT), it is observed that these are substantially the same between the variables “Rounds 1 to 5” and “Rounds Last 5”. Also, the average of goals per offensive transition is over or nearly 1 goal per game which demonstrates the impact of Offensive transitions has in today’s football. Being temporally closer to the pre-season period (“Rounds 1 to 5”) there is the possibility of influencing the values obtained. This is the optimal time for teams to establish and organize themselves according to solid game principles, tactical plans, and strategic and action guidelines to be used during the competitive period [22]. Those facts, highlights the importance in maintaining game patterns, during the more intense competitive period the team can maintain the same behaviors even though less training units are performed. The pre-competitive period has a concrete importance in defining the game patterns that last throughout the entire season. However, the data obtained indicate the constancy of the results happen indifferently of the variable consider and the championship. However, it would be expected throughout the competitive period, once the training process presupposes a systematic application, to ensure that adaptations occur [23], as well as periodization, to optimize competitive performance [24] and for that reason unexpectable the results showed residual differences on the “rounds last 5” variable.

The goals obtained through positive outcome (POS OUT), being goals from Set Pieces that resulted from an offensive transition, are as expected in smaller numbers than others. However, they have an important preponderance as these, along with the goals from Offensive Transition, are responsible for a high percentage of all goals obtained in the sample (Emerging Leagues 43.65%; Marginal Leagues 41.97%; Top Leagues 54.21%). This data unambiguously demonstrates that offensive transitions have greater chances of success than other styles of attack [8–10, 25]. The goals obtained exclusively through POS OUT remain virtually unchanged in the analyzed variables. This indicates that as the competition progresses, regardless the pre-analyzes games, teams aren’t able to avoid positive outcomes, particularly in high-risk areas. The Top Leagues present in more than half of the goals scored the participation of offensive transitions (either directly or through positive outcomes). This number, in addition to being expressive, has been growing as shown by previous studies where offensive transitions were responsible for 20 to 32% of all goals.

There were no differences found in goals scored from set pieces (SP) between the two groups of variables, “Rounds 1 to 5” and “Rounds Last 5”. However, it is important to note that in top leagues, SP goals have an average of more than one goal per game, indicating that stronger teams take better advantage of these moments. Top leagues attach significant importance to these moments as they have fewer training units and present less physical wear and tear in their preparation, enabling shorter and more specific training sessions, including individualized training for players [26].

Despite the various constraints, one would expect a significant increase in the number of goals scored during the competitive period due to the formation of game automatisms and normalization by the teams, or due to the differences between the teams and the willingness to take risks to achieve a higher number of goals [26]. The breaks in the competitive calendar may hinder the formation of the teams’ game automatisms [27]. Many players are involved in other competitions, including playing/training for national teams. However, it is the athletes and teams that are deprived of competition that suffer the most from the stagnation of the automatisms, which could be better honed in the context of actual competition.

The teams often have analysts responsible for analyzing the game patterns of opponents, which increases the predictability of their opponents’ actions [28]. It is therefore expected that teams will concede fewer goals from No Transitions (NT) in the “Rounds Last 5” variable, since these attacks take longer and allow more time for the teams to organize themselves defensively. Empirically, it is also expected that teams will score fewer goals through Positive Outcomes (POS OUT) during this period, as teams should be able to better control where offensive transitions end due to the presence of specialists in executing these moments. At the beginning of the competitive period (“Rounds 1 to 5”), one would expect more goals to be scored through Offensive Transitions (OT), Positive Outcomes (POS OUT), and Set Pieces (SP) due to the surprise factor and risk acceptance that teams tend to have in the early stages of the championships, where many points are at stake.

External factors that were not taken into account or recorded in this research include, but are not limited to, temperatures, the number of spectators present, the quality of the opponent, the number of training units, or the recovery times between competitions.

This study is groundbreaking as it compares two distinct competitive periods across three different groups of leagues. Future research could investigate the performance of teams in repeated matches to determine whether the observed differences are specific to certain leagues or if they can be generalized and compared. Additionally, exploring potential trends in end-of-season matches could yield valuable insights. It would also be beneficial to incorporate the perspectives of coaches and players, particularly in regard to the emphasis placed on goal-scoring strategies during the training microcycle.

## Conclusion

The aim of this study delved into a comparative analysis of goal-scoring methods across distinct phases of football championships, specifically examining the first five rounds (“Rounds 1 to 5”) and the last five rounds prior to the midway point through the season (“Rounds last 5”). The overarching finding reveals a general stability in goal-scoring strategies across most leagues. However, a notable exception emerged in Emerging Leagues, where the goals categorized as No-Transition (NT) exhibited variability during these specific competitive periods. Furthermore, the study emphasized the strategic significance of direct offensive transitions and positive outcomes, particularly in Top Leagues. This suggests that teams operating in these leagues benefit from prioritizing these approaches to enhance their goal-scoring potential. The conclusion underscores a universal and clear recommendation for teams, irrespective of their league categorization, to prioritize effective game patterns, with a particular emphasis on direct offensive transitions and positive outcomes.

Despite the valuable insights gained from this research, certain limitations should be acknowledged. The study could have delved deeper into the variations observed in No-Transition (NT) goals in Emerging Leagues. A more nuanced exploration of the factors contributing to the variability in goal-scoring methods during specific competitive periods within these leagues would enhance the comprehensiveness of the findings. Also, while highlighting the importance of defensive aspects, the study falls short of providing an in-depth analysis of these elements. A more detailed examination of the defensive strategies and challenges that teams face during different phases of the season would provide a more comprehensive understanding of goal-scoring dynamics.

The authors of this research recommend in future investigation a more extensive investigations into the variations in No-Transition (NT) goals in Emerging Leagues. Researchers are encouraged to explore the specific tactical, strategic, or contextual factors that contribute to the observed variability, providing a more granular understanding of goal-scoring patterns in these leagues. Also, future studies should focus on a more intricate examination of defensive aspects across all league groups. This could involve a detailed analysis of defensive strategies adopted by teams during different competitive periods, shedding light on the factors influencing defensive stability or vulnerability.

In sum, this research provides valuable insights into the consistency and nuances of goal-scoring methods, urging teams to align their game patterns for sustained football success strategically.

### Electronic supplementary material

Below is the link to the electronic supplementary material.

Supplementary Material 1

## Data Availability

The datasets used and/or analysed during the current study are available from the corresponding author on reasonable request.

## Abbreviations

TL: Top Leagues
ML: Marginal Leagues
EL: Emerging Leagues
OT: Offensive Transition
NT: No transition
SP: Set Pieces
POS OUT: Positive Outcome
OT + POS OUT: Goals obtained by Offensive transition and Positive Outcome
M: Media of these events per game
S: standard deviation
N: Total number of events
