# Supplementary material for: An analysis of transition-resulted goal scoring patterns in football leagues: a comparison of the first 5 rounds and the last 5 rounds prior midway of the season
Source: BMC Sports Sci Med Rehabil. 2024 Mar 2;16:60. doi: 10.1186/s13102-024-00854-0 (PMC10908095; doi:10.1186/s13102-024-00854-0)
Supplement: Supplementary file 1 — Supplementary Material 1 [file 13102_2024_854_MOESM1_ESM.docx]

Supplementary Information

For

“An analysis of transition-resulted goal scoring patterns in football leagues: a comparison of the first 5 rounds and the last 5 rounds”

By

Pedro Eusebio, Pablo Prieto-González, Rui Marcelino

Corresponding author: eusebio.pedro@gmail.com

1. Supplementary Information:

Supplementary information with the results obtained in each of the observed leagues is presented below:

Figure S1: Estimate Independent Mean Difference – Goals by OT – Comparison between “Rounds 1 to 5” and “Rounds Last 5”


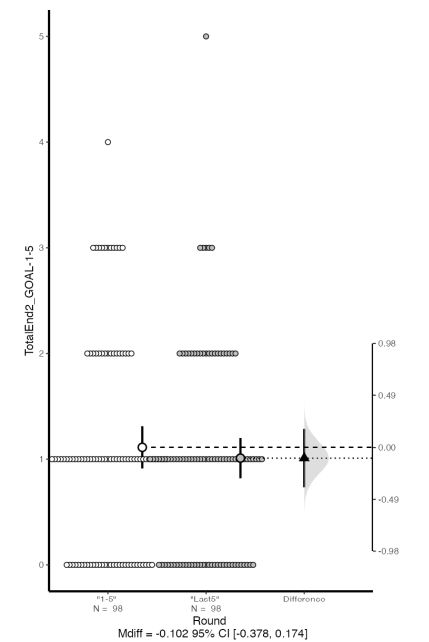


Emerging Leagues – Goal by OT


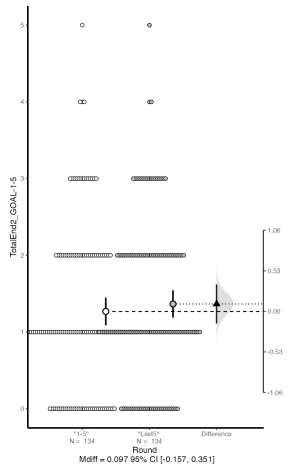


Top Leagues – Goal by OT


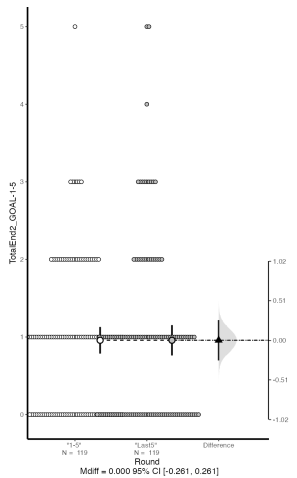


Marginal Leagues – Goal by OT

Note: Mdif: Mean difference; CI: Coefficient Interval; TotalEnd2_Goal 1-5: Total of Goals by Offensive Transition “Rounds 1 to 5” and “Last 5 Rounds”

Figure S2: Estimate Independent Mean Difference – Goals by NT – Comparison between “Rounds 1 to 5” and “Rounds Last 5”


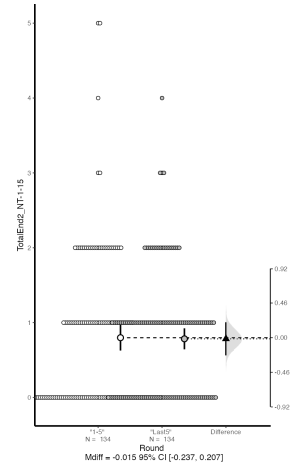


Top Leagues – Goal by NT


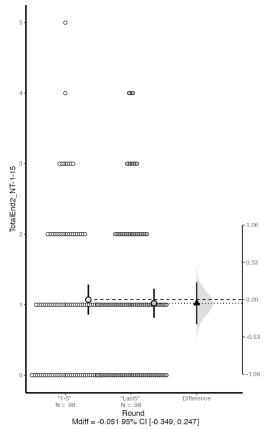


Emerging Leagues – Goal by NT

Marginal Leagues – Goal by NT


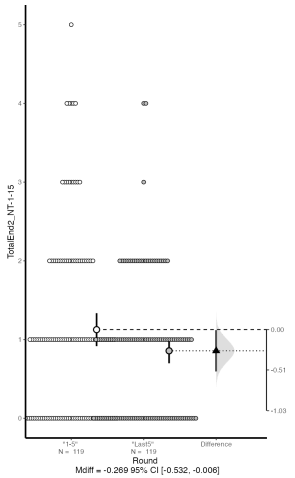


Note: Mdif: Mean difference; CI: Coefficient Interval; TotalEnd2_Goal 1-5: Total of Goals by No Transition “Rounds 1 to 5” and “Last 5 Rounds”

Figure S3: Estimate Independent Mean Difference – Goals by POS OUT – Comparison between “Rounds 1 to 5” and “Rounds Last 5”

Marginal Leagues – Goal by POS OUT


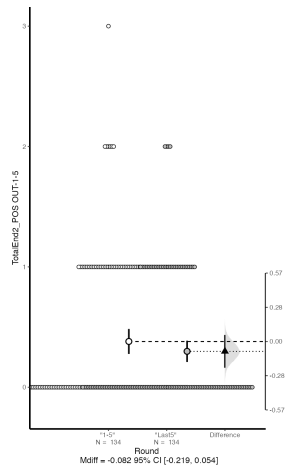


Top Leagues – Goal by POS OUT


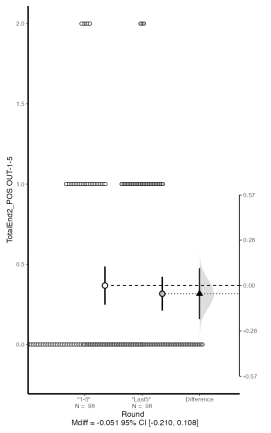


Emerging Leagues – Goal by POS OUT


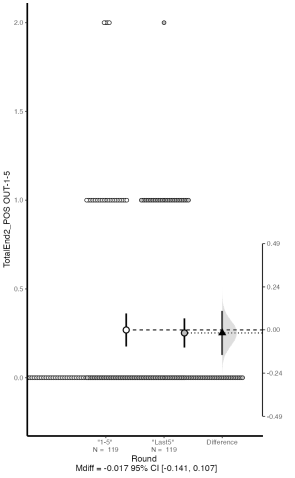


Note: Mdif: Mean difference; CI: Coefficient Interval; TotalEnd2_Goal 1-5: Total of Goals by Positive Outcomes “Rounds 1 to 5” and “Last 5 Rounds”

Figure S4: Estimate Independent Mean Difference –Goals by SP – Comparison between “Rounds 1 to 5” and “Rounds Last 5”


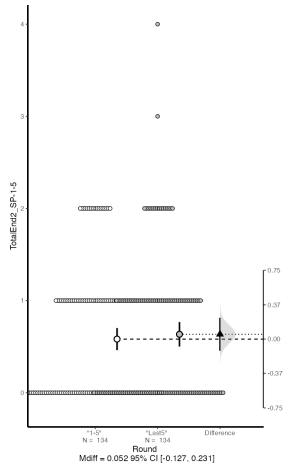


Top Leagues – Goal by SP


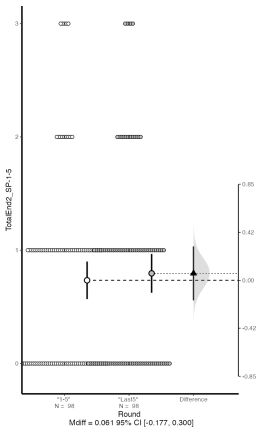


Emerging Leagues – Goal by SP


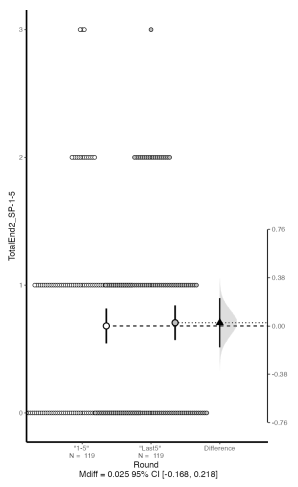


Marginal Leagues – Goal by SP

Note: Mdif: Mean difference; CI: Coefficient Interval; TotalEnd2_Goal 1-5: Total of Goals by Set Pieces “Rounds 1 to 5” and “Last 5 Rounds”

Supplementary information with the results obtained in each of the observed leagues is presented below:

Table S1: Distribution of the types of goals per leagues on the variables “Rounds 1 to 5” and “Rounds Last 5”.

|  |  | | Rounds - 1 to 5 | | | | Rounds - Last 5 | | | |  |
| --- | --- | --- | --- | --- | --- | --- | --- | --- | --- | --- | --- |
|  | **Games Observed with goals** | **Total Goals** | **Goals by NT** | **Goals by OT** | **Goals by SP** | **Goals by POS OUT** | **Goals by NT** | **Goals by OT** | **Goals by SP** | **Goals by POS OUT** | **% Of goals By OT + POS OUT** |
| Qatar | 58 | 189 | 33 | 37 | 24 | 7 | 26 | 28 | 20 | 14 | 45,50% |
| Saudi | 72 | 218 | 29 | 39 | 24 | 14 | 40 | 35 | 27 | 10 | 44,95% |
| UAE | 66 | 223 | 43 | 33 | 24 | 15 | 34 | 36 | 31 | 7 | 40,80% |
| Portugal | 78 | 223 | 40 | 40 | 27 | 14 | 34 | 33 | 21 | 14 | 45,29% |
| Netherland | 86 | 281 | 59 | 42 | 34 | 10 | 44 | 54 | 28 | 10 | 41,28% |
| Russia | 74 | 187 | 35 | 32 | 20 | 8 | 24 | 27 | 35 | 6 | 39,04% |
| Germany | 86 | 281 | 39 | 65 | 29 | 13 | 29 | 65 | 26 | 15 | 56,35% |
| Italy | 94 | 284 | 33 | 54 | 29 | 25 | 44 | 59 | 26 | 14 | 53,52% |
| Spain | 88 | 254 | 35 | 51 | 20 | 13 | 32 | 59 | 33 | 11 | 52,76% |
| Totals | 702 | 2140 | 346 | 393 | 231 | 119 | 307 | 396 | 247 | 101 |  |

Note: NT – No Transition. OT – Offensive Transition. SP – Set Pieces. POS OUT – Positive Outcome.

OT + POS OUT – Goals obtained by Offensive transition and Positive Outcome.

Table S2: Estimate Ind. Mean Difference – Goals obtained by OT per league – Comparison between “Rounds 1 to 5” and “Rounds Last 5”.

| Goals by OT |  | Rounds 1 to 5 | Rounds Last 5 | d_unbiased_ |
| --- | --- | --- | --- | --- |
| Emerging Leagues | UAE League | 1.00 (0.65; 1.35) 33 | 1.09 (0.72; 1.46) 33 | 0.09 (-0,40; 0,58) |
|  | Saudi League | 1.08 (0.77; 1.41) 36 | 0.97 (0.68;1.26) 36 | -0,12 (-0.60; 0.35) |
|  | Qatar League | 1.28 (0.87; 1.69) 29 | 0.97 (0.61; 1.32) 29 | -0.30 (-0.84; 0.22) |
| Marginal Leagues | Netherlands League | 0.98 (0.70; 1.25) 43 | 1.26 (0.91; 1.61) 43 | 0.27 (-0.16; 0.70) |
|  | Portugal League | 1.03 (0.67; 1.38) 39 | 0.85 (0.50; 1.19) 39 | -0.16 (-0.62; 0,29) |
|  | Russia League | 0.87 (0.60; 1.13) 37 | 0.73 (0.42; 1.04) 37 | -0.15 (-0.62; 0.31) |
| Top Leagues | German League | 1.51 (1.20; 1.83) 43 | 1.51 (1.14; 1.89) 43 | 0.00 (-0,43; 0,43) |
|  | Italy League | 1.15 (0.86; 1.44) 47 | 1.26 (0.98; 1.54) 47 | 0.11 (-0,30; 0,52) |
|  | Spain League | 1.16 (0.82; 1.50) 44 | 1.34 (1.06; 1.62) 44 | 0.17 (-0,25; 0,60) |

Note: M – Media of these events per game; S - standard deviation; 95% CI - confidence intervals (95%); N – Total number of events

Table S3: – Estimate Ind. Mean Difference – Goals obtained by NT per league – Comparison between “Rounds 1 to 5” and “Rounds Last 5”.

| Goals by NT |  | Rounds 1 to 5 | Rounds Last 5 | d_unbiased_ |
| --- | --- | --- | --- | --- |
| Emerging Leagues | UAE League | 1.37 (0.97; 1.78) 33 | 1.02 (0.76; 1.29) 33 | -0.31 (-0,75; 0,12) |
|  | Saudi League | 1.03 (0.67; 1.38) 36 | 0.87 (0.55; 1.19) 36 | -0.14 (-0,60; 0,30) |
|  | Qatar League | 0.95 (0.63; 1.26) 29 | 0.65 (0.43; 0.87) 33 | -0.35 (-0,83; 0,10) |
| Marginal Leagues | Netherlands League | 0.46 (0.22; 0.69) 43 | 0.21 (0.07; 0.36) 43 | -0,43 (-0,94; 0,05) |
|  | Portugal League | 0.81 (0.51; 1.10) 39 | 1.11 (0.73; 1.49) 39 | 0.30 (-0,17; 0,78) |
|  | Russia League | 1.14 (0.65; 1.62) 37 | 0.90 (0.53; 1.26) 37 | -0.21 (-0,74; 0,31) |
| Top Leagues | German League | 0.91 (0.56; 1.26) 43 | 0.67 (0.45; 0.90) 43 | 0.24 (-0,67; 0,19) |
|  | Italy League | 0.70 (0.45; 0.95) 47 | 0.94 (0.69; 1.18) 47 | 0.27 (-0,13; 0,69) |
|  | Spain League | 0.80 (0.49; 1.10) 44 | 0.73 (0.47; 0.99) 44 | -0.07 (-0,50; 0,35) |

Note: M – Media of these events per game; S - standard deviation; 95% CI - confidence intervals (95%); N – Total number of events

Table S4: – Estimate Ind. Mean Difference – Goals obtained by POS OUT per league – Comparison between “Rounds 1 to 5” and “Rounds Last 5”.

| Goals by POS OUT |  | Rounds 1 to 5 | Rounds Last 5 | d_unbiased_ |
| --- | --- | --- | --- | --- |
| Emerging Leagues | UAE League | 0.46 (0.22; 0.69) 33 | 0.21 (0.07; 0.36) 33 | -0.43 (-0,94; 0,05) |
|  | Saudi League | 0.39 (0.19; 0.59) 36 | 0.28 (0.13; 0.43) 36 | -0,21 (-0,68; 0,26) |
|  | Qatar League | 0.24 (0.05; 0.43) 29 | 0.48 (0.23; 0.74) 29 | 0.39 (-0,12; 0,94) |
| Marginal Leagues | Netherlands League | 0.23 (0.07; 0.39) 43 | 0.23 (0.10; 0.36) 43 | 0.00 (-0,43; 0,43) |
|  | Portugal League | 0.36 (0.19; 0.53) 39 | 0.36 (0.19; 0.53) 39 | -0.00 (-0,45; 0,45) |
|  | Russia League | 0.22 (0.06; 0.37) 37 | 0.16 (0.04; 0.29) 37 | -0.12 (-0,59; 0,34) |
| Top Leagues | German League | 0.30 (0.13; 0.47) 43 | 0.35 (0.18; 0.52) 43 | 0.08 (-0,35; 0,51) |
|  | Italy League | 0.53 (0.35; 0.71) 47 | 0.30 (0.14; 0.46) 47 | -0.40 (-0,82; 0,01) |
|  | Spain League | 0.25 (0.12; 0.38) 44 | 0.30 (0.11; 0.49) 44 | -0.08 (-0,51; 0,34) |

Note: M – Media of these events per game; S - standard deviation; 95% CI - confidence intervals (95%); N – Total number of events

Table S5: Estimate Ind. Mean Difference – Goals obtained by SP per league – Comparison between “Rounds 1 to 5” and “Rounds Last 5”.

| Goals by SP |  | Rounds 1 to 5 | Rounds Last 5 | d_unbiased_ |
| --- | --- | --- | --- | --- |
| Emerging Leagues | UAE League | 0.73 (0.39; 1.07) 33 | 0.94 (0.62; 1.26) 33 | 0.22 (-0,27; 0,72) |
|  | Saudi League | 0.67 (0.43; 0.91) 36 | 0.75 (0.46; 1.04) 36 | 0.10 (-0,36; 0,58) |
|  | Qatar League | 0.83 (0.53; 1.13) 29 | 0.69 (0.41; 0.97) 29 | -0,17 (-0,71; 0,35) |
| Marginal Leagues | Netherlands League | 0.79 (0.57; 1.02) 43 | 0.65 (0.42; 0.88) 43 | -0.19 (-0,62; 0,24) |
|  | Portugal League | 0.69 (0.43; 0.96) 39 | 0.54 (0.35; 0.73) 39 | -0.21 (-0,67; 0,24) |
|  | Russia League | 0.54 (0.31; 0.77) 37 | 0.95 (0.67; 1.22) 37 | -0.52 (0,06; 1,01) |
| Top Leagues | German League | 0.67 (0.46; 0.89) 43 | 0.60 (0.37; 0.85) 43 | -0.09 (-0,52; 0,33) |
|  | Italy League | 0.62 (0.40; 0.84) 47 | 0.55 (0.35; 0.76) 47 | -0.09 (-0,50; 0,32) |
|  | Spain League | 0.46 (0.27; 0.64) 44 | 0.75 (0.50; 1.00) 44 | 0.40 (-0,02; 0,83) |

Note: M – Media of these events per game; S - standard deviation; 95% CI - confidence intervals (95%); N – Total number of events

Supplementary information with the results obtained in each of the observed leagues is presented below:

Figure S5: Estimate Independent Mean Difference – Goals by OT – Comparison between “Rounds 1 to 5” and “Rounds Last 5”

Saudi League

Portugal League

Italy League


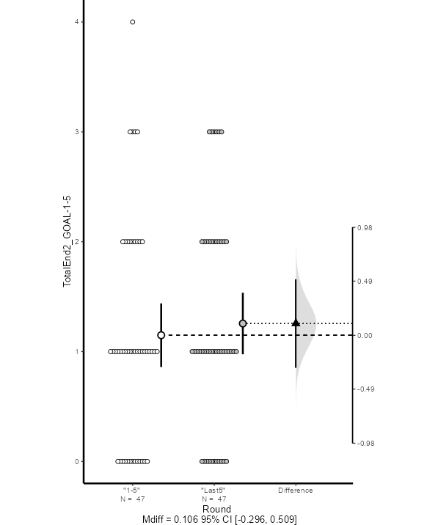

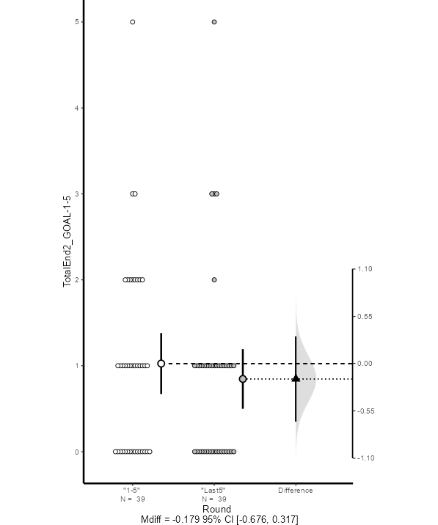

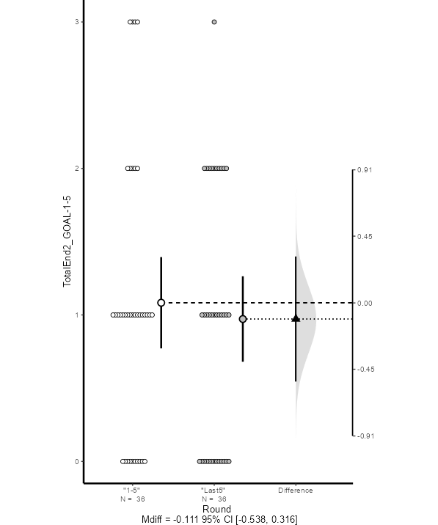


Russia League

Spain League

Qatar League


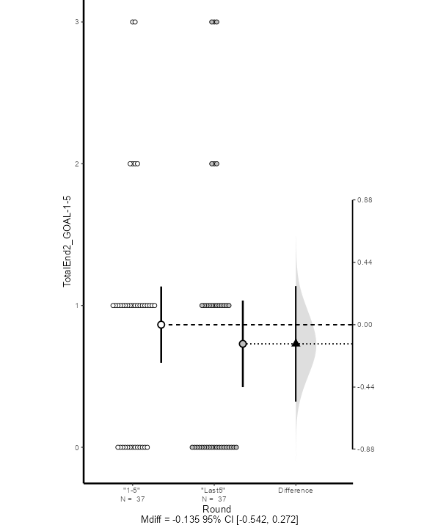

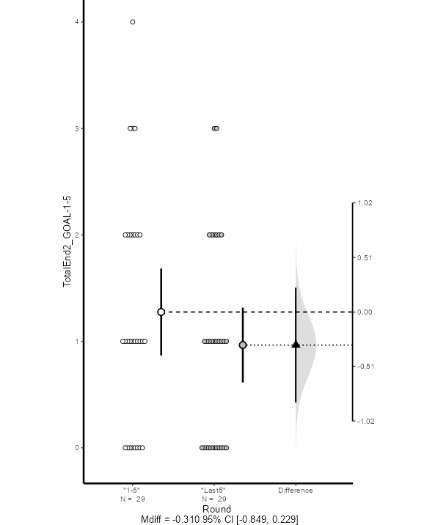

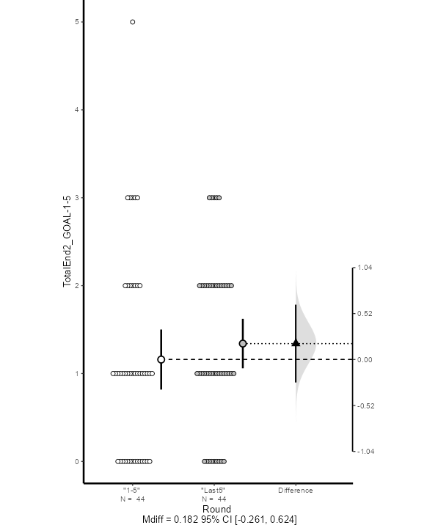


Netherlands League

German League

UAE League


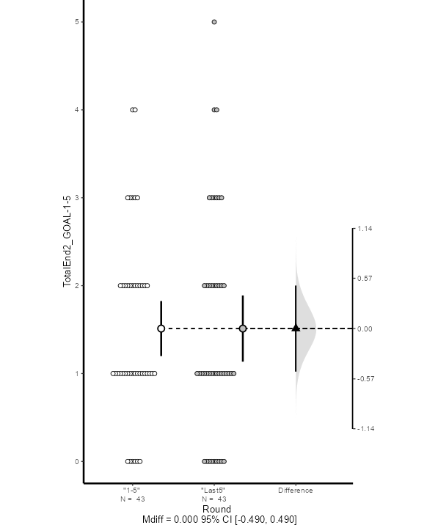

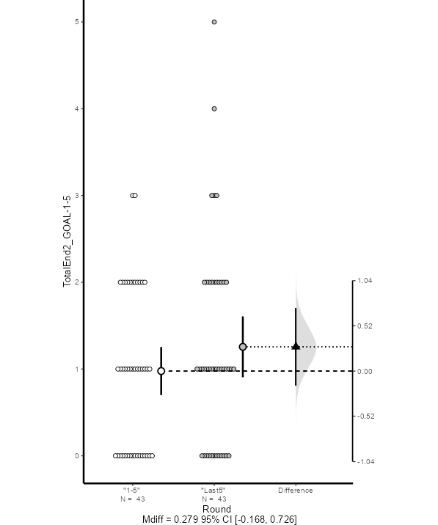

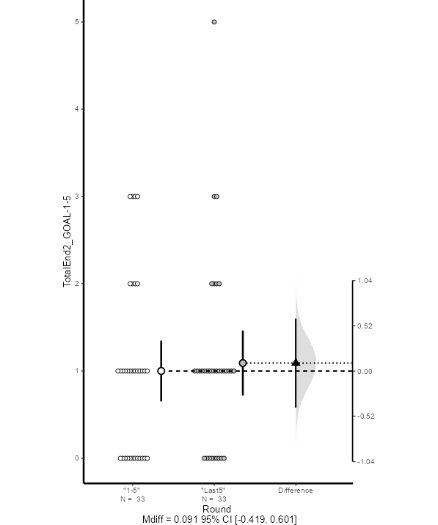


Note: Mdif: Mean difference; CI: Coefficient Interval; TotalEnd2_Goal 1-5: Total of Goals by Offensive Transition “Rounds 1 to 5” and “Last 5 Rounds”

Figure S6: Estimate Independent Mean Difference – Goals by NT – Comparison between “Rounds 1 to 5” and “Rounds Last 5”

Saudi League

Portugal League

Italy League


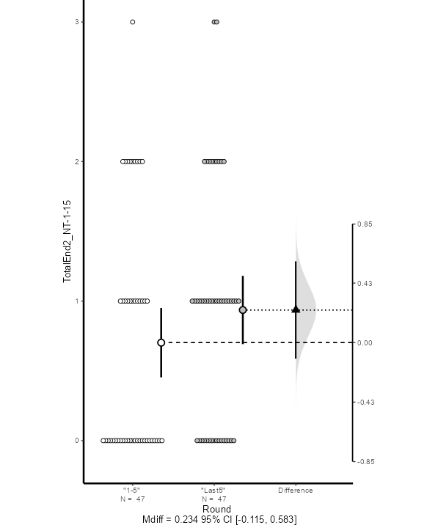

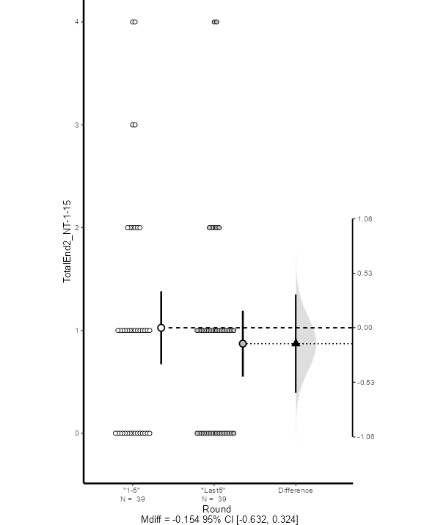

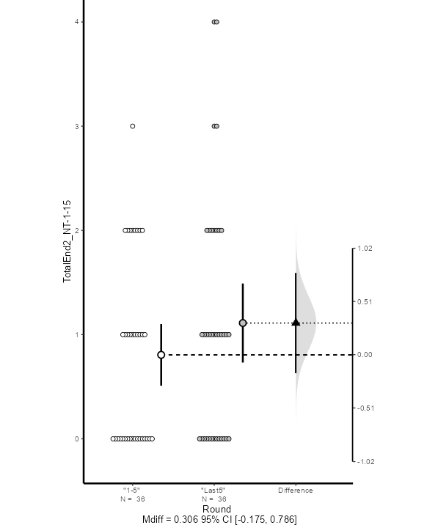


Russia League

Spain League

Qatar League


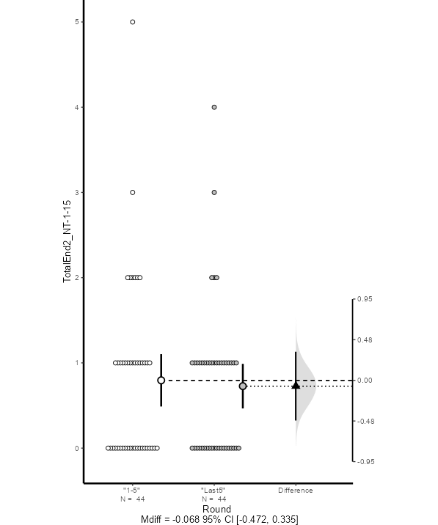

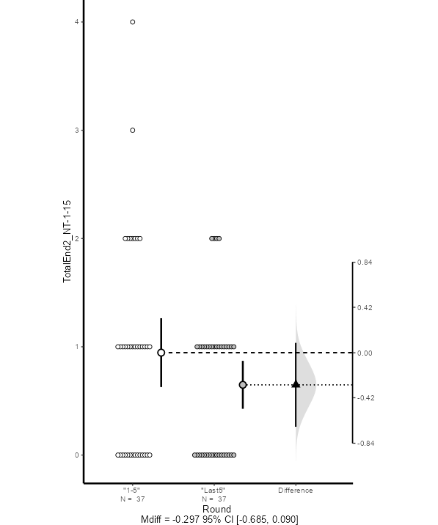

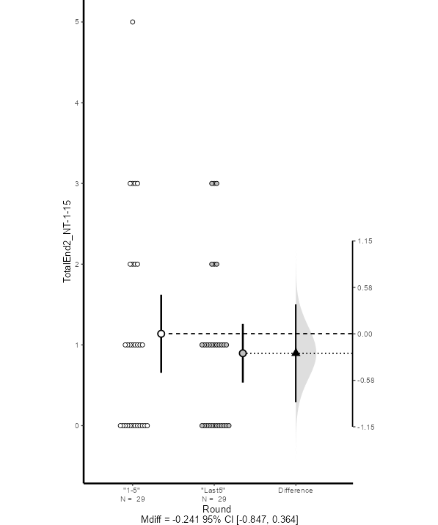


Netherlands League

German League

UAE League


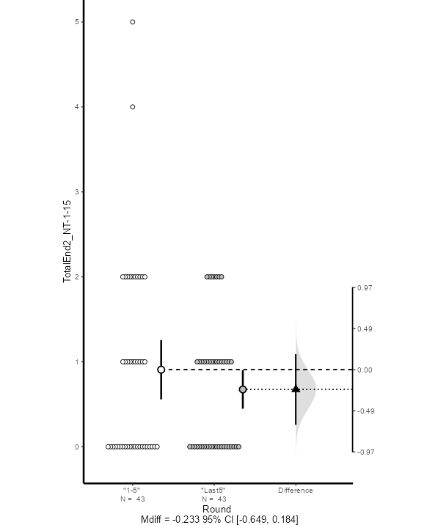

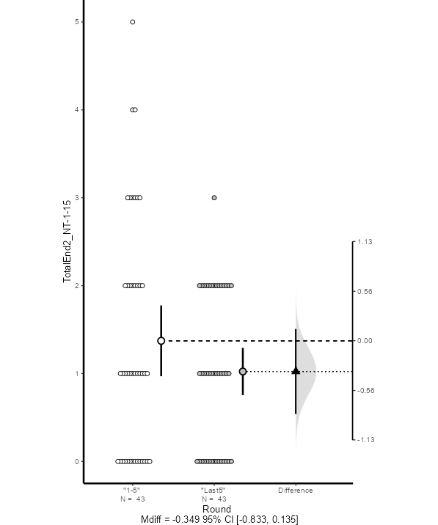

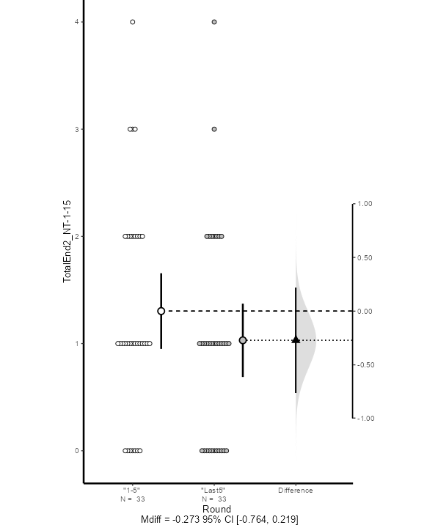


Note: Mdif: Mean difference; CI: Coefficient Interval; TotalEnd2_Goal 1-5: Total of Goals by No Transition “Rounds 1 to 5” and “Last 5 Rounds”

Figure S7: Estimate Independent Mean Difference – Goals by POS OUT – Comparison between “Rounds 1 to 5” and “Rounds Last 5”

Saudi League

Portugal League

Italy League


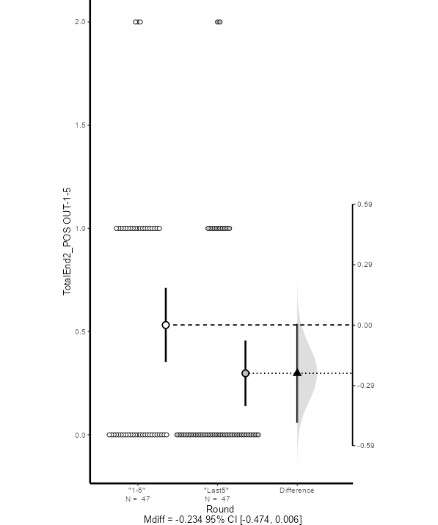

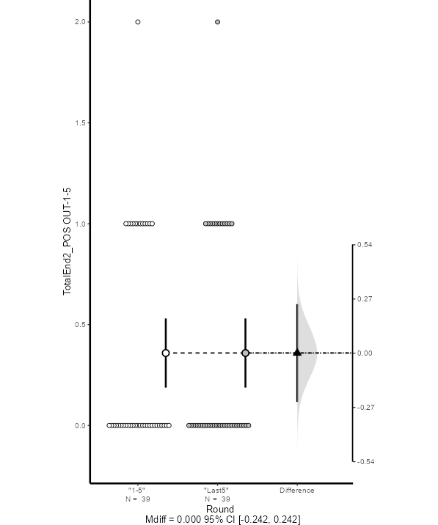

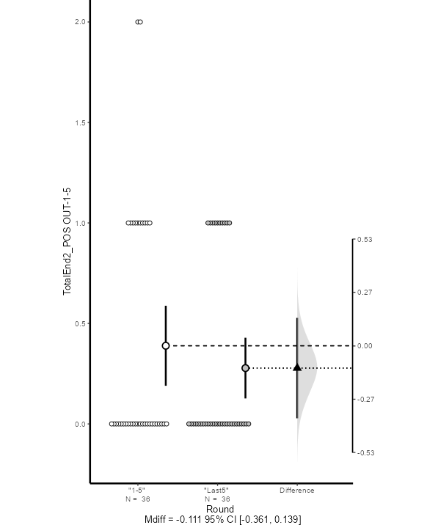


Netherlands League

German League

UAE League


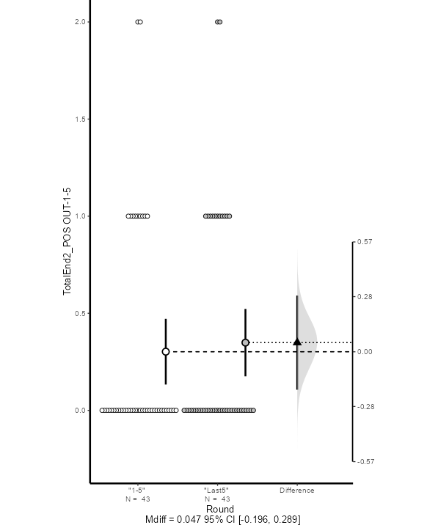

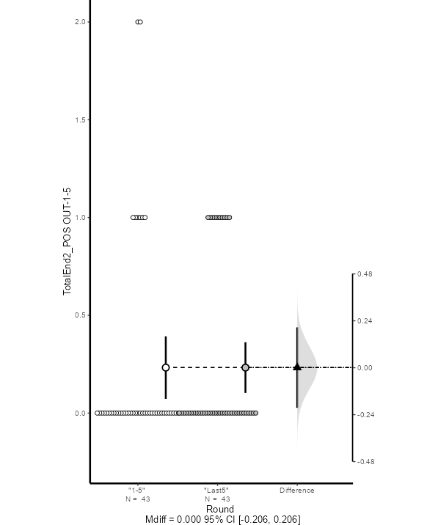

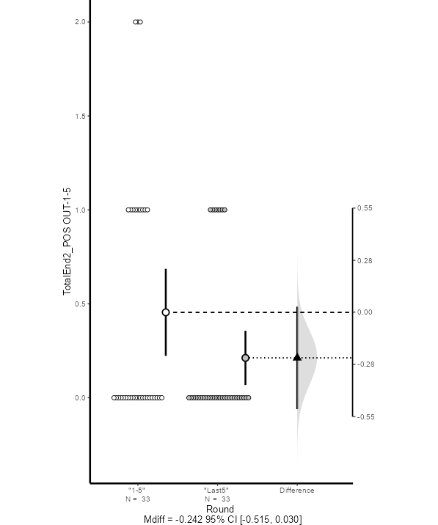


Russia League

Spain League

Qatar League


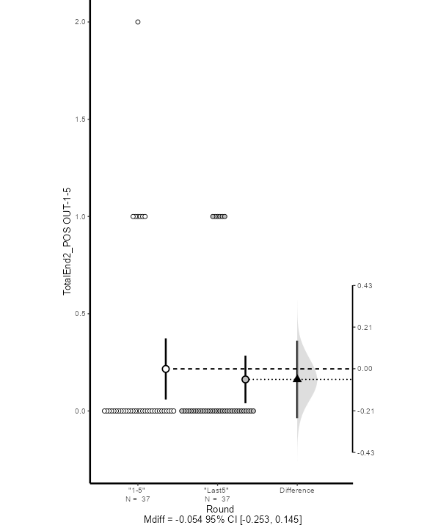

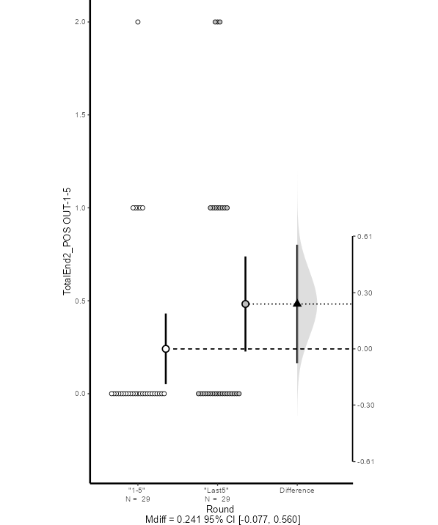

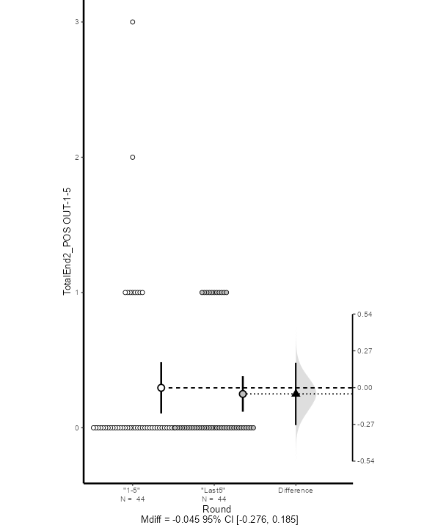


Note: Mdif: Mean difference; CI: Coefficient Interval; TotalEnd2_Goal 1-5: Total of Goals by Positive Outcome “Rounds 1 to 5” and “Last 5 Rounds”

Figure S8: Estimate Independent Mean Difference – Goals by SP – Comparison between “Rounds 1 to 5” and “Rounds Last

Saudi League

Portugal League

Italy League


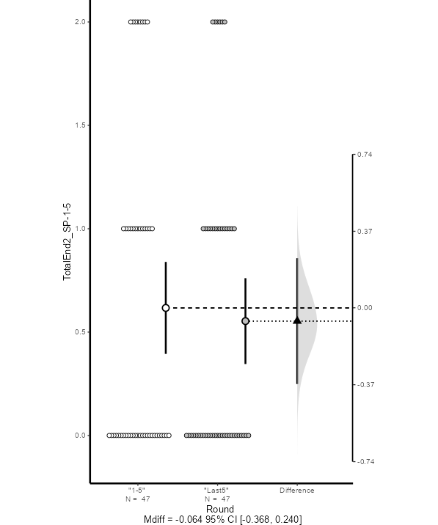

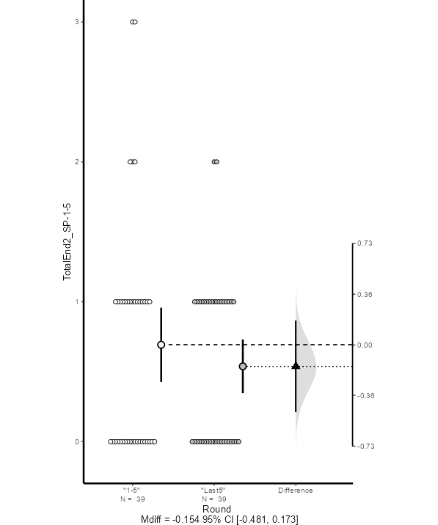

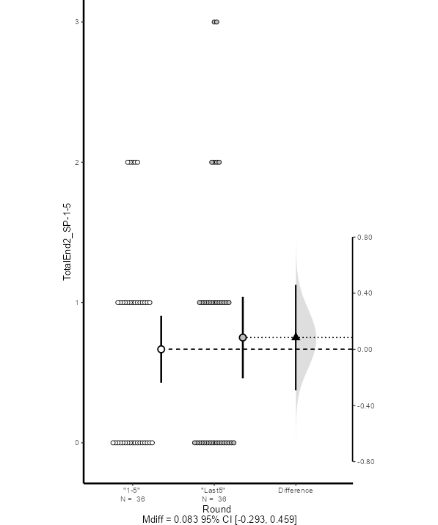


Russia League

Spain League

Qatar League


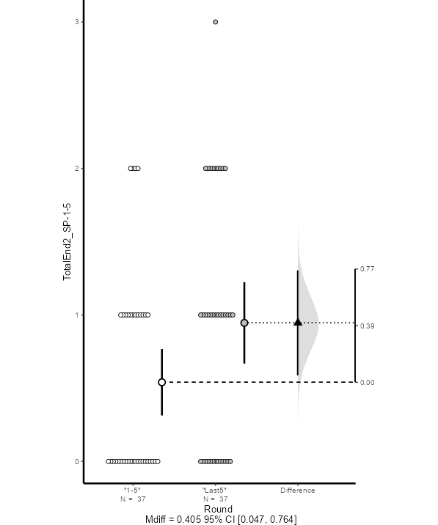

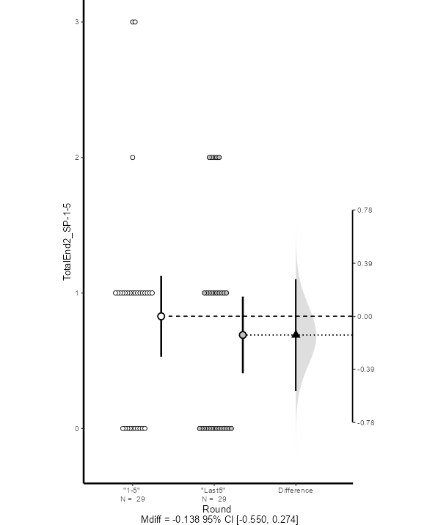

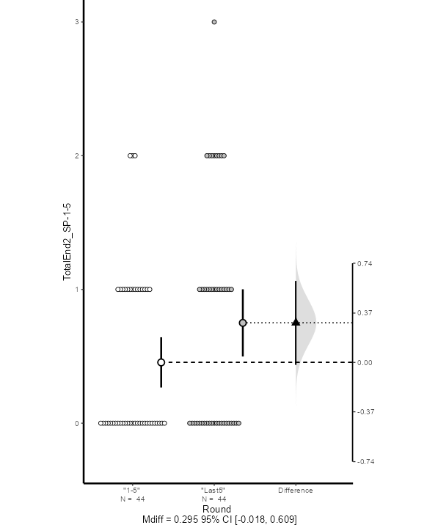


Netherlands League

German League

UAE League


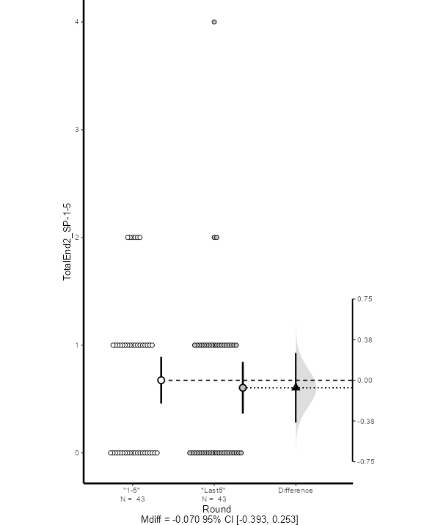

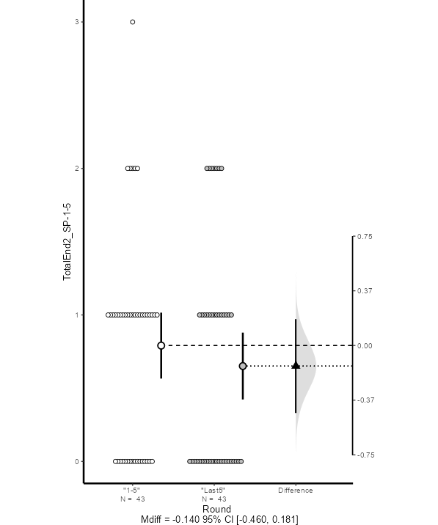

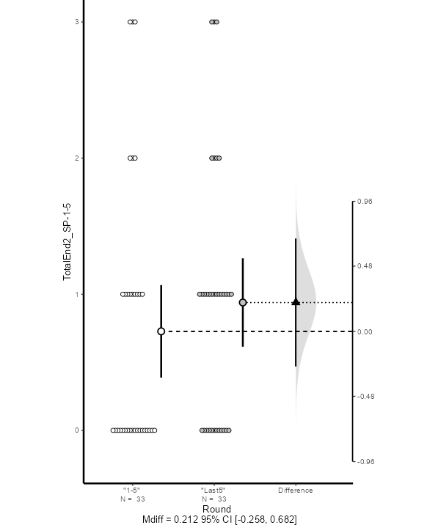


Note: Mdif: Mean difference; CI: Coefficient Interval; TotalEnd2_Goal 1-5: Total of Goals by Set Pieces “Rounds 1 to 5” and “Last 5 Rounds”
